# A line of zebrafish with development of abnormal spinal curvatures

**DOI:** 10.1186/1748-7161-9-S1-O44

**Published:** 2014-12-04

**Authors:** Henry Tomasiewicz, John Thometz, XueCheng Liu, Channing Tassone, Paula North

**Affiliations:** 1 University of Wisconsin-Milwaukee, Milwaukee, WI, USA; 2 Medical College of WI, Milwaukee, WI, USA

## Background

The lack of a good animal model system has hindered studying the etiology of idiopathic scoliosis. Recently, it has become clear that several fish species appear to exhibit spinal curvatures.

## Aims

To determine if the affected tissue in a line of zebrafish with spinal deformities resembles the pathology observed in pediatric populations with scoliosis.

## Design

This case series study was approved by IACUC.

## Methods

Potential founder fish with spinal curvatures were outcrossed with a wild type zebrafish line (AB) and the resulting siblings (F1 generation) crossed and the offspring (F2 generation) examined for signs of spinal curvature beginning at 14 days post fertilization (dpf). Spinal curvatures of the affected fish were visualized using either a Faxitron or by Alizarian red staining of the skeletons and the curvature measured from the resulting images in the thoracic, thoracolumbar, or lumbar regions. Affected and normal zebrafish were fixed, embedded, section and stained with hemotoxylin and eosin.

## Results

The degrees of curvatures ranged from 18° to 40° Histological data demonstrated structural changes as compared to normal fish spine. Out of 212 individuals in the F2 generation 28, or 13.2%, were observed to have spinal deformities at 21 dpf. Importantly, we did not observe spinal deformities in the F1 generation fish and similar age wild type fish, indicating the observed spinal deformities were due to a recessive mutation(s).

## Conclusion

An increase of scoliosis in family members and the occurrence of abnormal spinal curvatures in twins suggest a polygenetic inheritance pattern. We have noticed several fish in our zebrafish colony with spinal curvatures reminiscent of human idiopathic scoliosis. We suspect that “scoliosis” in these zebrafish results from mutation(s) in the zebrafish genome. And these fish can be the source of a zebrafish line in which the offspring exhibit a predictable frequency of scoliosis which can be used to study the etiology and progression of scoliosis.

